# Sugar-Sweetened Beverage Intake among Chilean Preschoolers and Adolescents in 2016: A Cross-Sectional Analysis

**DOI:** 10.3390/nu10111767

**Published:** 2018-11-15

**Authors:** Michael Essman, Barry M. Popkin, Camila Corvalán, Marcela Reyes, Lindsey Smith Taillie

**Affiliations:** 1 Department of Nutrition, Gillings School of Global Public Health, University of North Carolina at Chapel Hill, Chapel Hill, NC 27599-7400, USA; essmanm@live.unc.edu (M.E.); popkin@unc.edu (B.M.P.); 2 Carolina Population Center, University of North Carolina at Chapel Hill, Chapel Hill, NC 27516-2524, USA; 3 Institute of Nutrition and Food Technology (INTA), University of Chile, Santiago 7830490, Chile; ccorvalan@inta.uchile.cl (C.C.); mreyes@inta.uchile.cl (M.R.)

**Keywords:** beverage intake, children, adolescents, sugar-sweetened beverages, juice, milk

## Abstract

Background: Chile has the highest sugar-sweetened beverage (SSB) sales of any country and a growing burden of childhood obesity. This study examines SSB intake in Chilean children after a 5% SSB tax increase in 2014 but prior to marketing, labeling, and school policies implemented in 2016. Methods: 24-h recalls were collected in 2016 from two cohorts comprised of preschoolers 3–5 years of age (*n* = 961) and adolescents 12–14 years of age (*n* = 770) from low–moderate income neighborhoods. Beverages were categorized as regulated or unregulated according to whether they exceeded nutrient thresholds established by the 2016 policies. Results: Preschoolers consumed mainly beverage calories from regulated dairy beverages and substitutes (109 kcal, SD 30), unregulated dairy beverages (102 kcal, SD 24), and regulated fruit and vegetables drinks (44 kcal, SD 20). For adolescents, the greatest contributions came from regulated sodas (77 kcal, SD 47), regulated dairy beverages and substitutes (41 kcal, SD 16), and unregulated coffee and tea (41 kcal, SD 11). Overall, regulated beverages provided a greater proportion of calories than unregulated for preschoolers (15.0% vs. 11.8%) and for adolescents (9.1% vs. 5.0%). Conclusions: Before major policy implementation, regulated beverages accounted for a higher percentage of energy intake than unregulated beverages among both age groups. Future research will be needed to evaluate the impact of Chile’s new policies on sugary beverage intake in children.

## 1. Introduction

Chile has undergone a rapid increase in industrialization over the last 50 years and has achieved a high rating by the Human Development Index [1]. These economic changes have been linked with a nutrition transition [2], as the prevalence of stunting in Chile has greatly declined [3] while the prevalence of childhood obesity more than doubled between 1987 and 2000 [4]. Data from 2015 suggest as many as a third of middle school children are overweight [5]. Similar to the rest of the Latin American region [6,7], rapid increases in SSB intake have occurred concomitantly with the increases in overweight and obesity. Per capita SSB sales in Chile is the highest of any country (188 kilocalories per capita per day), and Chile also experienced the fastest absolute growth in SSB sales of any country in the world from 2009–2014 (+31 kilocalories per capita per day) [8]. This is a serious public health concern for Chile as SSBs are associated with obesity, diabetes, and cardiovascular disease risk [9].

In response to the growing prevalence of obesity, the Chilean government has implemented a number of policies to reduce SSB consumption along with other unhealthy ultra-processed foods among children. First, in October 2014, the government increased its tax on high-sugar beverages from 13% to 18% and decreased its tax on lower-sugar beverages from 13% to 10%. Recent evaluations of this tax found that household purchases of high-sugar beverages decreased about 3% after the tax, and that higher-income households showed greater declines [10]. Second, in June 2016, the Chilean government implemented marketing and labeling regulations on all foods and beverages containing added sugar, sodium, or saturated fat that exceed nutrient thresholds (Table S1). Specifically, the marketing regulations restricted the use of child-directed marketing techniques (i.e., the use of characters, movie tie-ins, or other techniques that uniquely appeal to children) and also restricted the advertisement of regulated products on programs and websites with a high proportion of child viewers. The regulations also prohibited the sales and promotion of regulated products in schools [11]. Packaged products that exceed the nutrient thresholds are required to carry a warning label (an octagon containing the words “high in” sugar, sodium, saturated fat, or calories) to clearly inform consumers of the high energy or nutrient density. While the initial nutrient thresholds were implemented in 2016, the Chilean nutrient thresholds become increasingly strict over time to allow industry manufacturers to reformulate the sugar content of beverage products. The regulations have three phases of implementation, with each phase lowering the critical nutrient thresholds for regulation (Table S1). The third and final phase of regulations will be implemented in June 2019.

Despite the rapid implementation of these policies to reduce SSB intake, levels of SSB consumption among children and adolescents after the tax but before the marketing, labeling, and school regulations is unknown. Understanding Chilean children’s SSB intakes prior to the implementation of these policies is important as a baseline measurement to which future changes in intake can be compared. Second, and more generally, understanding SSB intakes in childhood and adolescence is especially important, as SSB intake in these groups is associated with obesity and greater adult weight gain later in life [12,13,14].

The objectives of this study were to examine SSB intake in two cohorts of Chilean preschoolers and adolescents using data from 24-h recalls collected in 2016, after the SSB tax but before the marketing, labeling, and school restrictions, and to determine the proportion of beverages which would be regulated under the new Chilean policies. Aggregate sales data from Euromonitor International were also provided to present national-level beverage sales trends. 

## 2. Materials and Methods

### 2.1. Study Design and Sample

Data were obtained in 2016 from two longitudinal cohort studies: the Food Environment Chilean Cohort (FEChiC) which consists of children between 3 to 5 years of age and the Growth and Obesity Chilean Cohort Study (GOCS) which consists of adolescents now 12 to 14 years of age; both cohorts represent low–middle income Chilean population. 

The Growth and Obesity Chilean Cohort Study (GOCS) began in 2006 as part of a collaboration between the Chilean National Preschool Program (JUNJI) and the Chilean National School Board Program (JUNAEB). Children recruited to participate in the study were between the ages of 2.6 and 4.0 years in that year and attended the kindergartens of six counties (“comunas”) in the South-East area of Santiago, Chile. The cohort has since grown to 26 counties in Santiago, and approximately two thirds of the children are from South-East Santiago. Children were invited to participate if they were singleton births, had a birth weight between 2500 and 4500 g, and did not have any physical and psychological conditions that could affect their growth. Further details on recruitment have been previously described [15]. 

FEChiC is a new cohort that began in 2016. Children and their mothers were recruited using a similar approach as the GOCS participants, by working in partnership with JUNAEB, from 15 public nursery schools in the southeast area of Santiago, Chile. Children from participating schools were invited to join the cohort if they were single births and did not have any physical and psychological conditions that could affect their growth. Inclusion criteria required that they be singleton births, that they did not have any mental disabilities, and they were free from gastrointestinal diseases that could affect dietary habits and behaviors. In addition, mothers were required to be the main person responsible for feeding their children to ensure they could provide accurate information about their children’s dietary intake. Informed consent was obtained for both cohorts from the parents or guardians of the children. Study protocols for both cohorts were approved by the Ethics Committee of the Institute of Nutrition and Food Technology (INTA).

### 2.2. Inclusion/Exclusion Criteria

#### 2.2.1. FEChiC

From the FEChiC cohort (3 to 5 years of age), dietary intake information was collected from 961 children. Three of the children were excluded for having implausible 24-h dietary recalls, defined as a total energy intake below 400 kilocalories (kcal) per day. After exclusions, there were 958 children 3 to 5 years of age in the final baseline sample for 2016.

#### 2.2.2. GOCS

From the GOCS cohort (12 to 14 years of age), dietary data were collected from 768 adolescents. One adolescent was excluded for an implausible 24-h dietary recall, which reported a total energy intake of zero kilocalories. After this exclusion, 767 adolescents 12 to 14 years of age were included in the analysis.

### 2.3. Assessment of Dietary Intake

Dietary data were collected by trained nutritionists following a standardized 24-h recall method during scheduled visits of participants to CEDINTA (diagnostic center) during 2016. For preschool children, mothers primarily reported their child’s dietary intake, and the children helped assist with any missing details. To assist with estimation of portion size, the dietary assessment uses the Photographic Atlas of Chilean Food and Typical Preparations [16], and the School Feeding Program (PAE) draft of JUNAEB. The collection process is performed using the 5-step multiple pass method assisted by software developed for this purpose (SER 24) following the USDA’s multiple-pass method [17]. The software links food and beverage names reported in dietary recalls to nutrient profile data in the USDA Food Composition Databases [18]. If a Chilean beverage did not exist in the USDA Food Composition Databases, then the closest possible match was selected on the basis of the most similar nutrient profile. 

In 20% of the sample, a second recall was collected within the next month to assess variability. In cases where there were two dietary recalls, the recall rated as more reliable was chosen. This was determined according to whether it was a special occasion (such as a birthday or party), reported as eating more or less than usual, or rated by the nutritionist administering the recall as having low reliability. If neither of the days was rated as having lower reliability, the first recall day was chosen for consistency. 

### 2.4. Beverage Classification System

To group beverages, we used a system previously developed to classify SSBs in the US [19] and Mexico [20], but adapted to the Chilean context. This allows us to compare beverage categories across countries while also ensuring that the beverage groups reflect Chilean’s typical beverage consumption patterns and that nutritionally similar beverages are grouped together. We classified beverages into seven broad categories: (1) Water (bottled or tap) and flavored water; (2) energy and sport drinks; (3) sodas; (4) fruit and vegetable juice drinks; (5) coffee and tea; (6) dairy based beverages and dairy substitutes (will be referred to as “dairy and substitutes”); (7) special formula and nutritional supplements. The water category contains tap, purified, or bottled water as well as industrialized flavored waters. For waters, coffee, and tea, only those which are ready to drink and include added sugar can be subject to regulations because addition of sugar after purchases was not considered in the regulation. Special formula and nutritional supplements (SFNS) include all infant, toddler (e.g., Pediasure), maternal, elderly (e.g., Ensure) special milks and nutritional supplements intended to meet the nutritional requirements for that time of life. Dairy and substitutes includes milk modifiers such as Nesquik, Milo, and Cola Cao. Table S2 contains descriptions of each beverage analysis category.

Each of these categories was further designated as either regulated or unregulated according to the critical nutrient thresholds in Chilean law, making a total of 14 categories into which all beverages were included. The term regulated refers to those products that would be considered regulated under the first phase of the Chilean law implemented in 2016. These beverages would be banned from marketing to children, from being sold in school, and, if packaged, would receive a front-of-package warning label(s). These products had either added sugars, sodium, or saturated fats and exceed the thresholds for kilocalories (kcal), sodium, saturated fats, or sugars of the first phase of the law (Table S1). In addition, the nutrition thresholds become stronger over time, with more stringent thresholds implemented in 2018 and 2019. Thus, we also conducted analyses in which we assigned regulation status using the 2019 nutrition criteria in order to understand the proportion of beverages in our sample that would be regulated under the final phase of the regulation. Beverages that did not have added sugars, sodium, or saturated fats or did not exceed these thresholds were placed in the “unregulated” category in this analysis. This applied to beverages such as 100% juice, which contained sugars but no added sugars, and was therefore classified as unregulated. For beverages, sugar was the most common nutrient or ingredient that caused a beverage to exceed thresholds and be classified as regulated. Given that our main results refer to the regulation status of beverages that were consumed before the 2016 regulations were implemented, we will interpret our results as reflective of beverages that would be subject to regulations once they were implemented later that year. 

Powdered beverages were reconstituted to reflect actual intake, based on being mixed with water. Powdered beverages included: powdered milk, coffee, tea, industrialized flavored waters prepared from powder, and milk modifiers. To reconstitute, first all powders were identified (e.g., “leche en polvo” is powdered milk). Next, if there was a liquid present at the same meal, then the liquid was combined with the powder to represent the beverage as consumed. Estimates for coffee and tea consumption included sugar that was added at the time of consumption. However, coffees and teas that contained sugar added by the consumer were not considered a regulated beverage because sugar was added after the product was purchased. 

### 2.5. Sociodemographic Variables

Mother’s education was used as a proxy for socioeconomic status (SES) because we have found that mother’s education is a key determinant of a child’s diet. Mothers’ highest levels of education were categorized into three groups: less than high school complete, high school complete, and more than high school (i.e., the mother is either in pursuit of or has completed educational training beyond high school). Other SES related variables available in the data included home ownership and car ownership.

### 2.6. Beverage Sales Data

Data on beverage volume sales from Chile were retrieved from the Passport Global Market of Euromonitor International from 2003–2017 to provide context to the overall beverage consumption patterns at the national level in Chile over the past decade [21]. Euromonitor mainly collects data on major brands and retail sales, acquired from the major global and national beverage companies. This includes key brands but does not include small local companies. All sales are updated at the end of the year and represent aggregate sales data for both on-trade and off-trade sales for the entire country of Chile. Off-trade sales are those that occur in retail locations such as supermarkets, and on-trade sales occur in bars, restaurants, and cafes. Previous work has discussed these methods in greater detail [8]. Beverage classification groups used by Euromonitor International [21] are provided in Table S3.

For overall sales of regular and low calorie sodas versus bottled water, we analyzed sales trends of three main groups: total bottled water, SSBs, and low calorie (diet) cola carbonates. The category SSBs includes regular cola carbonates, non-cola carbonates, liquid concentrates, powder concentrates, juice drinks up to 24% juice, nectars (25–99% juice), ready-to-drink (RTD) coffee, RTD tea, and sports and energy drinks. 100% juice was analyzed as a separate group as it does not involve added sugar. Volume sales reported are total volume sales, which includes both on-trade and off-trade sources. Trends in beverage sales are displayed for SSBs versus diet carbonates and bottled waters, followed by trends in SSB categories other than carbonated sodas. 

### 2.7. Statistical Analysis

All analyses were performed using Stata 15 (StataCorp, College Station, TX, USA). Sociodemographic characteristics of the two study populations, including BMI-for-age *z*-score were originally tabulated in Stata and presented in Table 1. To describe beverage intake, mean volume (mL) and energy (kilocalories) intake per capita and per consumer for each beverage category were calculated using multiple linear regression models adjusted means for sex, age, mother’s education, total energy intake, and day of intake (weekday/weekend) in each age group (children 3 to 5 years of age and adolescents 12 to 14 years of age). We performed a sensitivity analysis that examined whether there were differences in mean intake for any beverage categories comparing weekdays versus weekends. We found that some groups had statistically significant differences in mean intakes, and as a result of this sensitivity analysis, models included a binary variable to adjust for whether dietary recall was collected on a weekday versus a weekend. Separate linear regression models were used to test for differences by sex and mother’s education, with beverage intake category (i.e., regulated or unregulated beverages) as the dependent variable. Total energy was controlled for in analyses that measured absolute intakes to ensure that differences in beverages intakes were not due to differences in total dietary energy intake. Graphs of results from Euromonitor were generated using Microsoft Excel version 16.16.3 (Microsoft, King County, WA, USA) after annual beverage sales data were retrieved from the Passport Global Market of Euromonitor International [21].

## 3. Results

### 3.1. National-Level Beverage Sales Volume

**Figure 1:**
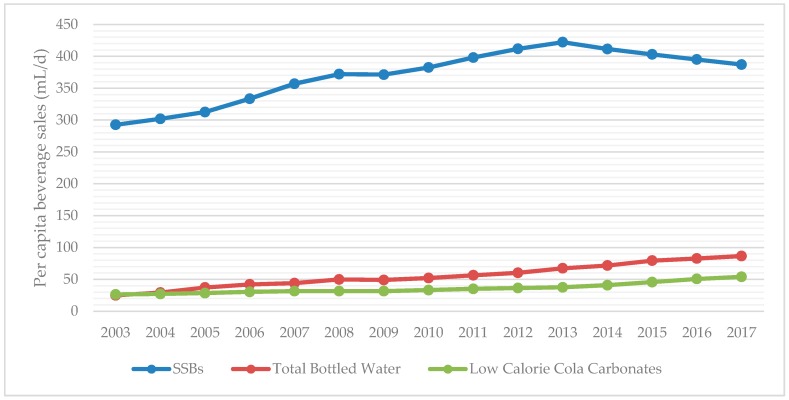
Trends in SSB, diet cola carbonates, and bottled water sales in Chile from 2003–2017. SSBs include categories of regular cola carbonates, non-cola carbonates, liquid concentrates, powder concentrates, juice drinks of <99% juice, sport and energy drinks, and ready-to-drink tea. All data were derived from the Passport Global Market of Euromonitor International. SSB, sugar-sweetened beverages.

**Figure 2:**
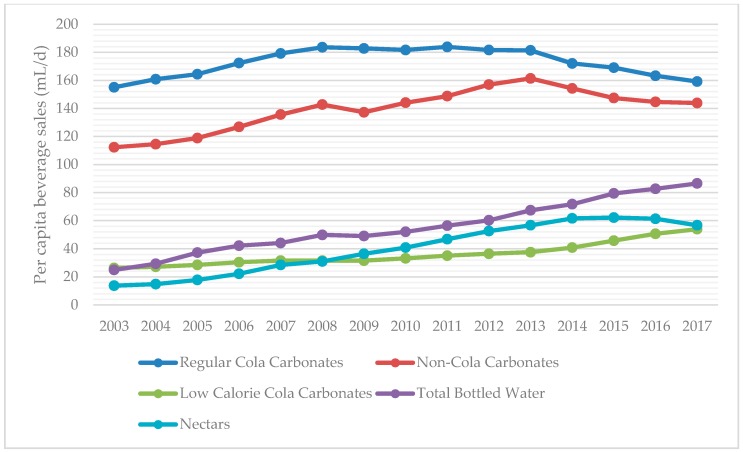
Sales trends for three largest SSB categories (Regular Cola Carbonates, Non-Cola Carbonates, and Nectars) compared to bottled water and diet cola carbonates. All data were derived from the Passport Global Market of Euromonitor International.

**Figure 3:**
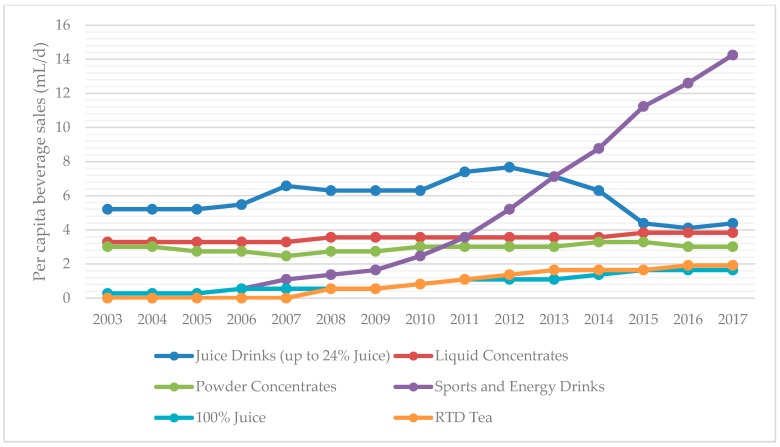
Trends in sales of SSB categories excluding sodas and nectars (the two largest categories) in Chile from 2003–2017. All data were derived from the Passport Global Market of Euromonitor International. RTD, ready-to-drink.

According to data from Euromonitor Passport International, SSB sales declined 8.2% (35 mL per capita per day) from 2013–2017 (Figure 1). The two largest SSB categories, cola and non-cola carbonates, account for most of the decline in SSB sales, while bottled waters and low calorie (diet) cola carbonates have increased (19 and 16 mL per capita per day, respectively) from 2013–2017 (Figure 2). However, SSBs still accounted for a vast proportion of the beverage sales in Chile according to available data from 2003–2017, especially compared to all bottled water products and low calorie (diet) carbonated products (Figure 1). Sugar-sweetened beverage sales are approximately seven times that of low calorie (diet) carbonates (387 vs. 54 mL per capita per day) as of 2017. Of all beverage categories for which Euromonitor collects data, sports and energy drinks was the fastest growing beverage category from 2003–2017 (Figure 3). Bottled water sales are growing more rapidly than either regular sugar or diet carbonated beverages, but they are still purchased at less than a quarter the amount of SSBs (Figure 1). 

### 3.2. Sociodemographic Characteristics of the Two Cohorts

The sociodemographic characteristics of the two cohorts in 2016 are presented in Table 1. A higher proportion of the mothers from the FEChiC cohort pursued or completed education beyond high school compared to the mothers from the GOCS cohort. 

### 3.3. Beverage Consumption Patterns by Type of Beverage in the Two Cohorts in 2016

**Figure 4:**
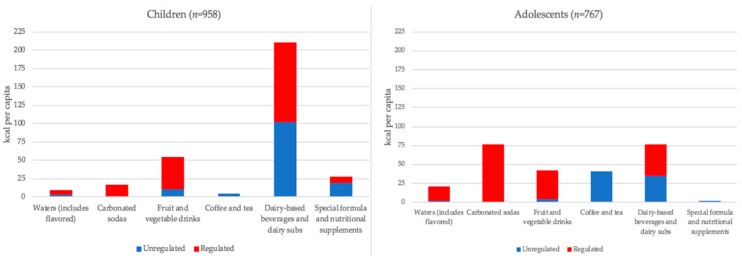
Energy consumption from beverages presented by beverage type and regulation status. Units are in average kilocalories (kcal) per capita per day. Red bars are regulated beverages and blue bars are unregulated beverages.

**Figure 5:**
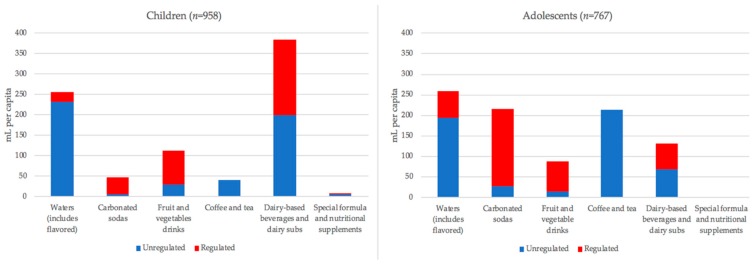
Beverage volume consumption presented by beverage type and regulation status. Units are in average milliliters (mL) per capita per day. Red bars are regulated beverages and blue bars are unregulated beverages.

Mean total beverage consumption accounted for 324 (SD 83) kcal per capita per day in the cohort of preschoolers 3 to 5 years of age (Figure 4; Table S4) and 261 (SD 85) kcal per capita per day in the cohort of adolescents 12 to 14 years of age (Figure 4; Table S5). The greatest beverage volumes consumed were from water and diet flavored waters, dairy-based beverages and dairy substitutes, and fruit and vegetable drinks for preschoolers and waters, sodas, and coffee for adolescents (Figure 5). Sports and energy drink consumption in both cohorts was negligible (Tables S4 and S5).

In preschoolers, the greatest contribution to beverage calories came from regulated dairy beverages and substitutes (109 kcal, SD 30), unregulated dairy beverages (102 kcal, SD 24), and regulated fruit and vegetable drinks (44 kcal, SD 20) (Figure 4; Table S4). Compared to preschoolers with mothers that completed high school, preschoolers with mothers who completed less than high school consumed significantly more regulated waters and industrial flavored waters (+5 kcal per capita, *p* < 0.01) and significantly more regulated fruit and vegetable drinks (10 kcal per capita, *p* < 0.05). Compared to preschoolers with mothers that completed high school, preschoolers with mothers who completed more than high school consumed significantly less regulated soda (8 kcal per capita, *p* < 0.05) and significantly more calories (9 kcal, *p* < 0.05) from special formula.

In adolescents 12 to 14 years of age, the greatest contribution of calories came from regulated carbonated sodas (77 kcal, SD 47), regulated dairy beverages and substitutes (41 kcal, SD 16), and unregulated coffee and tea (41 kcal, SD 11). Regulated fruit and vegetable drinks were the third highest contributor of calories from regulated beverages and the fourth highest category overall (Figure 4 and Table S5). The greatest beverage volumes consumed were from coffee and tea, water and diet flavored water, and regulated carbonated sodas (Figure 5; Table S6). Compared to adolescents with mothers who completed high school, adolescents with mothers who completed more than high school consumed significantly less regulated soda (42 kcal per capita, *p* < 0.01) and significantly fewer calories from unregulated coffee and tea (17 kcal per capita, *p* < 0.05).

### 3.4. Beverage Consumption Patterns Analyzed According to Regulation Status

These results can also be compared in terms of total regulated versus total unregulated beverage intake. Table 2 and Table 3 display the percent of total energy derived from regulated and unregulated beverages in each cohort, reported per capita and per consumer. In both cohorts, males and females consumed roughly the same percentage of total calories from regulated beverages, and in both groups regulated beverages contributed a greater share of total calories than unregulated beverages (Table 2 and Table 3). There were no differences in calories consumed from beverage types comparing males versus females, controlling for mother’s education and total energy intake (Table 2 and Table 3).

In preschoolers 3 to 5 years of age, beverages that would be regulated according to the first phase of Chilean law accounted for 15.0% (SD 13.2%) and unregulated beverages accounted for 11.8% (SD 11.6%) of total daily energy intake. In absolute terms, preschoolers consumed more calories from regulated beverages (185 kcal per capita) than from unregulated beverages (139 kcal per capita) (Table S4). Males consumed on average 16 more calories per capita from regulated beverages compared to females, but differences were not statistically significant. The vast majority of beverage calories consumed by preschoolers were from dairy drinks, approximately half of which are regulated (Figure 4; Table S4). When analyzed by consumer, regulated dairy based beverages and substitutes, unregulated dairy based beverages and substitutes, and regulated fruit and vegetable drinks contributed the highest caloric intakes (Table S4). There were no significant differences in the percentage of participants that consumed regulated beverages when differences were analyzed by mother’s education status in the 3 to 5 years of age cohort (Table 2). There were no significant sex differences or differences between mother’s education groups for total regulated or unregulated beverage consumption.

In adolescents 12 to 14 years of age, regulated beverages accounted for 9.2% (SD 8.5%) and unregulated beverages for 4.9% (SD 5.8%) of total energy. Adolescents consumed more calories from regulated beverages (174 kcal per capita) than from unregulated beverages (87 kcal per capita) (Table S5). Within the regulated beverage category, sodas, fruit and vegetable drinks, and dairy based beverages and substitutes were the greatest sources of calories (Figure 4; Table S5). Males consumed on average 48 more calories from regulated beverages compared to females, but did not differ when adjusted for total energy. There were differences by mother’s education in the older cohort: adolescents with mothers of the highest education category consumed a significantly lower proportion of their daily calories from regulated beverages compared to adolescents of mothers with the lowest education category (Table 3). Regulated dairy based beverages and substitutes, regulated fruit and vegetable drinks, and regulated sodas also contributed the most calories when non-consumers were excluded from analyses (Table S5). Among consumers of regulated beverages, adolescents with mothers in the highest education group consumed less regulated beverages than adolescents with mothers in the lowest education group (Table 3).

Compared to the June 2016 cutoffs, these data suggest the third phase regulations would capture 6.9% more calories from regulated beverages (205 vs. 185 kcal/capita) in preschoolers (Tables S4 and S7) and 10.8% more regulated beverage calories in adolescents (186 vs. 174 kcal/capita) (Tables S5 and S8). In terms of beverage volume, the third phase regulations would capture 11.4% more volume from regulated beverages (371 vs. 333 mL/capita) in preschoolers (Tables S9 and S10) and 6.2% more volume from regulated beverages (414 vs. 390 mL/capita) in adolescents (Tables S6 and S11).

### 3.5. Sensitivity Analysis

In a sensitivity analysis comparing dietary recalls collected on weekdays versus weekends, we found a greater intake of regulated sodas on weekends compared to weekdays in preschoolers (33 vs. 14 kcal/capita) and adolescents (116 vs. 66 kcal/capita) (Table S12). Preschoolers also consumed significantly more fruit and vegetable drinks on weekdays compared to weekends (48 vs. 18 kcal/capita) (Table S12).

## 4. Discussion

A key finding of this study is that there was a decline (8.2%) in per capita SSB consumption in Chile from 2013–2017. In absolute terms, since 2013, SSB sales have decreased 35 mL per capita per day while bottled waters and diet cola carbonates have increased 19 and 16 mL per capita per day, respectively. These amounts suggest that a small shift may be occurring in Chile away from some SSB sales which are being substituted for diet carbonates and bottled water products. These trends are in concordance with recent evidence that the tax on high-sugar SSBs implemented in October 2014 led to small decreases in purchases over the next year and increases in bottled water [10]. 

However, SSB consumption remains high, with SSB consumption approximately seven times that of diet carbonates and nearly five times that of bottled waters [21]. Overall, approximately a quarter of calories in the diets of the children and about a sixth in adolescent diets came from beverages. In children aged 4–6, the greatest contribution to beverage calories came from regulated dairy beverages and substitutes followed by unregulated dairy beverages and substitutes, and in adolescents 12 to 14 years of age the greatest contribution to beverage calories came from regulated carbonated sodas. 

A key objective of this study was to examine whether the beverages that children consumed in early 2016, prior to the implementation of the Chilean regulations on marketing and labeling in June 2016, would be subject to the regulation according to the 2016 nutrient criteria. The regulation was implemented in Chile at the end of June 2016, after which all regulated beverages were subject to marketing restrictions, banned from being sold in schools, and, if packaged, received a warning label. The timing of this analysis is important because it provides a baseline for understanding beverage consumption patterns in 2016 prior to the regulations, enabling future studies of how beverage intake changed after the regulations. Future studies will be needed to evaluate changes in SSB consumption as a result of the Chilean regulations. 

A key result of this study is that regulated beverages contributed more calories than unregulated beverages for preschoolers (185 kcal per capita from regulated compared to 139 kcal per capita from unregulated; Table S4) and for adolescents (174 kcal per capita from regulated compared to 87 kcal per capita from unregulated; Table S5). This is in concordance with previous work that found high sugary beverage consumption in Chilean children, including the national Food Consumption Survey In Chile (ENCA) 2010–2012, which found that 92% of Chilean school children consume some amount of sugar sweetened beverages daily, with a median intake of 424 mL [22].

As a baseline analysis, this study suggests that the new Chilean marketing and front-of-package (FOP) labeling regulations are targeting a large portion of beverage calories in the Chilean diet. Experimental settings of FOP labeling show that simple, clear labeling schemes are linked to increased understanding and use, particularly among those who are interested in healthy eating and losing weight [23,24]. The simplicity of nutrition labels has also associated with greater improvements in nutrition understanding among lower socioeconomic status groups compared to more complex labels [25], and overall FOP labels with a simple format are easier to evaluate than more complex ones [26]. This evidence suggests that the straightforward warning labels for the high critical nutrients in Chile may facilitate understanding and behavior change better than in other countries with more complex labels, but more studies will be needed to determine the effects of the package of regulations that were implemented in 2016. 

When comparing the consumption of total regulated versus total unregulated beverage categories, there were few differences in regulated or unregulated consumption when analyzed by sex or socioeconomic level, except for one finding of a lower regulated beverage consumption by adolescents with the highest educated mothers. This is a similar result to a study using data from ENCA 2010 that found higher consumption of certain SSB types in children from lower socioeconomic level households [22]. Male adolescents consumed more calories from regulated beverages compared to females, but calories from regulated beverages did not differ when adjusted for total energy, which suggests the greater regulated beverage consumption may be due to greater total energy intake and not differences in consumption patterns. This finding agrees with previous work that found no sex differences in SSB intake in Chilean schoolchildren [22].

One of the major findings is that dairy is consumed in high quantities, particularly in the younger cohort. This is important because so few countries have chosen to consider dairy products under the sugar-sweetened beverage category that most tax and regulate, including dairy drinks with added sugars. Although they remain untaxed also in Chile, dairy drinks with added sugar are regulated and will be subject to marketing restrictions, school sales restrictions, and front of package label warnings if they exceed nutrient levels established by the Chilean law. Subsequent evaluations of the Chilean regulations may provide a unique opportunity to understand one option for countries to use in addressing dairy products with excessive or any added sugar in the future.

Another interesting result of comparing Euromonitor data to the dietary intake data in these two cohorts is the difference in consumption of energy drinks in the children versus the sales of energy drinks in the general population. In relative terms, energy drinks are the fastest growing beverage category according to Euromonitor data, but intake remains negligible in children and small in adolescents. This presents an opportunity for prevention in the youth if prevention is possible before the youth begin to drink energy drinks in large amounts.

This study has several limitations. One limitation of this study is that the nutrient data associated with the standardized 24-h dietary recalls is linked with USDA nutrient data and not a Chilean food composition table. Although all food and beverage items have been matched with the best possible listing in the USDA food composition table, this is a source of measurement error in our results because there may be differences in nutrient composition for certain foods in the US and Chilean food supplies. However, despite these limitations, packaged beverages are more similar and easier to match than foods, which often require creating recipes to group individual ingredients. We have now collected nutrient data from more than 15,000 food products including beverages so future studies will be able to assess the potential impact of using USDA nutrient data. Other sources of measurement error are those inherent to using a 24-h recall to assess dietary intake. For example, even if the diet interview was deemed to be reflective of a typical day, people do not typically consume the same foods and beverages every single day, and therefore some participants may have been non-consumers of regulated beverages even if they typically consume them. Such episodically consumed foods may not be captured as well using a 24-h dietary recall versus other methods of diet assessment such as food frequency questionnaires. Although not the focus of this analysis, our data may have limited power to detect differences in estimated beverage intake between subgroups in our two cohorts. The small effect sizes and large standard errors reported in this study make it less likely to detect a statistically significant difference if one truly exists. To improve these limitations related to both repeated measures of dietary intake and study power, a larger national sample that is representative with repeated days of recall will be needed to understand usual intake and understand whether there are differences in sex and mother’s education or other measures of socioeconomic status. This would include a proportionate representative sample of weekends and holidays in order to understand usual intake of SSBs. In addition, methods such as the National Cancer Institute method for usual intake assessment could be adapted for use in this data. These data are also collected from children and adolescents from low and middle income households living in the South East of Santiago, the capital city of Chile. Therefore, these children may not be representative of the dietary habits of all children living in other regions of Santiago, and there may also be differences in SSB consumption for urban versus rural households that we are not able to determine with these data. 

The main strength of this study is that it provides a baseline measurement of SSB intake in Chilean children and adolescents after the 2014 SSB tax increase but before the regulations on marketing, labeling, and school sales went into effect in June 2016. This is important because baseline measurements will be needed to determine the effects of the marketing, labeling, and sales regulations that occurred after the SSB tax. The strength of including data from Euromonitor International in this paper is that it provides additional information about the national level beverage sales in Chile. Both of these sources of data will be valuable moving forward in evaluating the effects of the marketing, labeling, and sales restrictions on beverage intake in Chile.

## 5. Conclusions

In summary, the beverages categories that contributed the greatest share of calories to the diets of these two cohorts were regulated and unregulated dairy beverages and regulated fruit and vegetables drinks in preschoolers and regulated sodas and regulated dairy beverages in adolescents. The majority of beverage calories for both preschool children and adolescents that would be subject to the FOP and marketing regulations in its first phase if they are not reformulated. Future research will be needed to monitor the impact of Chile’s marketing, labeling, and school policies on regulated sugary beverage intake and subsequent obesity and cardiometabolic disease risk.

## Figures and Tables

**Table 1:** Socio-demographic characteristics of 3–5 year-olds in the 2016 wave of the FEChiC study and 12–14 year-old children in the 2016 wave of the GOCS study by sociodemographic information for respondents of the dietary intake survey.

	3–5 Year-Old Cohort (*n* = 958)	12–14 Year-Old Cohort (*n* = 767)
**Sex**	**%**	**%**
Male	48.5	48.4
Female	51.5	48.6
Missing		3.0
**Mother’s Education**		
Less than High School Complete	18.1	28.3
High School Complete	41.3	44.6
More than High School Complete	40.6	22.3
Missing		4.8
**Mother Works Outside Home**		
Yes	47.7	28.6
No	50.4	64.4
Sometimes	1.9	2.0
Missing		5.1
**Home Ownership**		
Yes	54.1	54.8
No	45.9	41.9
Missing		3.4
**Automobile Ownership**		
Yes	52.6	40.9
No	47.4	55.7
Missing		3.4
	**Mean (SD)**	**Mean (SD)**
**Age**	4.3 (0.5)	13.1 (0.5)
**BMI-for-Age *z*-Score**	1.04 (1.21)	0.95 (1.12)

**Table 2:** Beverage consumption per capita and per consumer reported as a percent of total energy intake and stratified by sociodemographic group in Chilean children (3 to 5 years of age).

	*n*	% Consuming Regulated Beverages (SD)	Total Energy Intake kcal (SD)	Total Calories from Beverages (SD) †	% of Total Energy from Regulated Beverages (SD)		% of Total Energy from Unregulated Beverages (SD)	
**Gender**			**Per Capita**	**Per Capita**	**Per Capita**	**Per Consumer**	**Per Capita**	**Per Consumer**
Male	465	80.0 (40.0)	1273 * (391)	327 (190)	14.5 (12.9)	18.2 (11.9)	11.4 (11.3)	12.2 (11.3)
Female	493	82.4 (38.2)	1197 * (386)	321 (180)	15.4 (13.6)	18.7 (12.7)	12.3 (12.0)	12.9 (11.9)
**Mother’s Education**								
Less than High School Complete	173	80.9 (39.4)	1205 (352)	308 (171)	14.5 (13.6)	17.9 (12.9)	11.5 (11.9)	12.3 (11.9)
High School Complete	396	81.3 (39.0)	1250 (414)	326 (181)	14.6 (13.2)	18.0 (12.4)	12.3 (12.1)	13.0 (12.1)
More than High School Complete	389	81.2 (39.1)	1230 (382)	329 (194)	15.5 (13.1)	19.0 (12.0)	11.5 (11.0)	12.2 (11.0)

* In testing gender differences, analyses controlled for mother’s education. In testing differences between groups by mother’s education, analyses controlled for sex. * denotes statistical significance at the *p* < 0.05 level. † Testing that compares absolute intakes are controlled for total energy.

**Table 3:** Beverage consumption per capita and per consumer reported as a percent of total energy intake and stratified by sociodemographic group in Chilean adolescents (12 to 14 years of age).

	*n*	% Consuming Regulated Beverages (SD)	Total Energy Intake kcal (SD)	Total Calories from Beverages (SD) †	% of Total Energy from Regulated Beverages (SD)		% of Total Energy from Unregulated Beverages (SD)	
**Gender**			**Per Capita**	**Per Capita**	**Per Capita**	**Per Consumer**	**Per Capita**	**Per Consumer**
Male	377	75.3	2008 * (649)	287 (97)	9.4 (9.4)	12.5 (7.8)	4.8 (5.6)	5.1 (5.7)
Female	375	74.9	1738 * (642)	235 (67)	8.9 (8.3)	11.8 (7.5)	5.1 (5.9)	5.4 (6.0)
**Mother’s Education**								
Less than High School Complete	217	74.7 (43.6)	1861 (684)	280 (202)	9.9 * (9.7)	13.3 * (9.0)	5.4 (6.3)	5.8 (6.3)
High School Complete	342	76.0 (42.8)	1884 (640)	260 (182)	9.2 (8.2)	12.1 (7.3)	4.7 (5.4)	5.0 (5.4)
More than High School Complete	171	70.2 (45.9)	1866 (670)	239 (170)	8.1 * (7.5)	11.5 * (6.4)	5.0 (6.2)	5.2 (6.3)
Missing	37							

* In testing gender differences, analyses controlled for mother’s education. In testing differences between groups by mother’s education, analyses controlled for sex. * denotes statistical significance at the *p* < 0.05 level. † Testing that compares absolute intakes are controlled for total energy.

## Supplementary Materials

The following are available online at http://www.mdpi.com/2072-6643/10/11/1767/s1, Table S1: Three phases of the Chilean law that sets threshold limits on energy, sodium, saturated fat, and total sugars [27]. Table S2. Beverage groups used for classification grouped by regulation status. Table S3. Beverage classification groups used by Euromonitor International [21]. Table S4. Consumption per capita and per consumer of beverage type for preschoolers (3–5 years) reported in kilocalories (kcal) (*N* = 958). Regulation status is assigned according to June 2016 cutoffs (first phase). Adjusted means and standard deviations (SD) are presented. Table S5. Consumption per capita and per consumer of beverage type for adolescents (12–14 years) reported in kilocalories (kcal) (*N* = 767). Regulation status is assigned according to June 2016 cutoffs (first phase). Adjusted means and standard deviations (SD) are presented. Table S6. Consumption per capita and per consumer of beverage type for adolescents (12–14 years) reported in mL (*N* = 767). Regulation status is assigned according to June 2016 cutoffs (first phase). Adjusted means and standard deviations (SD) are presented. Table S7. Consumption per capita and per consumer of beverage type for preschoolers (3–5 years) reported in kilocalories (kcal) (*N* = 958). Regulation status is assigned according to June 2019 cutoffs (third phase). Adjusted means and standard deviations (SD) are presented. Table S8. Consumption per capita and per consumer of beverage type for adolescents (12–14 years) reported in kilocalories (kcal) (*N* = 767). Regulation status is assigned according to June 2019 cutoffs (third phase). Adjusted means and standard deviations (SD) are presented. Table S9. Consumption per capita and per consumer of beverage type for preschoolers (3–5 years) reported in milliliters (mL) (*N* = 958). Regulation status is assigned according to June 2016 cutoffs (first phase). Adjusted means and standard deviations (SD) are presented. Table S10. Consumption per capita and per consumer of beverage type for preschoolers (3–5 years) reported in milliliters (mL) (*N* = 958). Regulation status is assigned according to June 2019 cutoffs (third phase). Adjusted means and standard deviations (SD) are presented. Table S11. Consumption per capita and per consumer of beverage type for adolescents (12–14 years) reported in mL (*N* = 767). Regulation status is assigned according to June 2019 cutoffs (third phase). Adjusted means and standard deviations (SD) are presented. Table S12. Sensitivity analysis presenting significant differences in energy consumed from beverage categories on weekdays versus weekends.
