# Supplementary material for: Sugar-Sweetened Beverage Intake among Chilean Preschoolers and Adolescents in 2016: A Cross-Sectional Analysis
Source: Nutrients. 2018 Nov 15;10(11):1767. doi: 10.3390/nu10111767 (PMC6265687; doi:10.3390/nu10111767)
Supplement: Supplementary file 1 [file nutrients-10-01767-s001.zip › nutrients-366391-SI.pdf]

Supplementary Materials

Table S1. Three phases of the Chilean law that sets threshold limits on energy, sodium, saturated fat, and total sugars [27].

Limits on contents for critical nutrients in beverages	Apply from 26th of June, 2016	Apply from 26th of June, 2018	Apply from 26th of June, 2019
Energy (kcal/100 ml)	100	80	70
Sodium (mg/100 ml)	100	100	100
Total sugar (g/100 ml)	6	5	5
Saturated fats (g/100 ml)	3	3	3

Table S2. Beverage groups used for classification grouped by regulation status.

**Beverage group name**	**Beverage group description**
Regulated	Meets at least one condition for regulation based on exceeding added levels of sugar, sodium, or saturated fat. For beverages, typically based on added sugar levels.
Sweetened flavored water	Includes industrialized flavored water with added sugar that crosses regulation thresholds.
Energy and sports drinks	Includes sport drinks and energy drinks that meet regulation conditions.
Carbonated sodas	Includes carbonated sodas that meet regulation conditions.
Fruit and vegetable drinks	Includes industrialized fruits and vegetables juices that meet regulation conditions.
Coffee and tea	Coffee beverages, including instant, drinks containing coffee with other ingredients such as milk or sugar, also includes coffee substitutes of plant origin (soy, chicory, barley, other) or ready-to-drink powder. Can only meet regulation status if crosses thresholds at purchase, not due to consumer adding sugar to prepared beverage.
Dairy based beverages and dairy substitutes	Includes milk, dairy drinks, drinkable yogurt, fermented milk, soy-based beverages, other dairy substitutes, and milk modifiers with sugar.
Special formulas and nutritional supplements	Includes all infant, toddler, maternal, elderly special milks and nutritional supplements intended to meet nutritional requirements for age/stage of life, with at least one of added sugar, sodium, or saturated fat passing the regulation thresholds.
Unregulated	Does not meet any conditions for regulation based on exceeding added levels of sugar, sodium, or saturated fat. For beverages, typically based on added sugar levels.
Water and diet flavored water	Includes plain water (tap or bottled) and industrialized flavored water without sugar crossing threshold.
Diet sports and energy drinks	Includes sport drinks and energy drinks that do not meet regulation conditions.
Carbonated diet sodas	Includes carbonated sodas that do not meet regulation status.
Dairy-based beverages and dairy substitutes (DBDS)	Includes milk, dairy drinks, drinkable yogurt, fermented milk, soy-based beverages, other dairy substitutes, and milk modifiers; plain and flavored without sugar.
100% Fruit/vegetable juice	Includes 100% fruit and vegetable juice without added sugar or other ingredients.
Coffee and tea	All coffee beverages, including instant, powder or granules, roasted, ground, drinks containing coffee with other ingredients such as milk or sugar, also includes coffee substitutes of plant origin (soy, chicory, barley, other); Includes regular and herbal teas, made from leaves or other plant parts, all forms including leaves, ground, prepared tea bags, to brew or ready-to-drink or powder.
Special formulas and nutritional supplements	Includes all infant, toddler, maternal, elderly special milks and nutritional supplements intended to meet nutritional requirements for age/stage of life that do not meet regulation conditions.

Table S3. Beverage classification groups used by Euromonitor International[21].

**Beverage group name**	**Beverage group description**
SSBs	Aggregate SSB category includes all select groups from below
Regular Cola Carbonates	Regular sugar colas with added caffeine that traditionally use vanilla and the kola nut for flavor. May include other flavors.
Lemonade/Lime	Both juice-based and non-juice-based lemon and lime flavored drinks
Ginger Ale	Carbonated beverage that contain ginger or flavoring. Branded examples include Canada Dry, Schweppes and Seagram's.
Tonic Water/Other Bitters	Carbonated beverage with quinone added. Also includes alcoholic carbonated bitters.
Orange Carbonates	Both juice-based and non-juice-based orange flavored drinks
Other Non-Cola Carbonates	Miscellaneous soft drinks that were not included in the above categories. Examples include Mountain Dew and Dr Pepper.
Liquid Concentrates	Mixed beverages typically based on flavored syrups. Often fruit flavors.

Powder Concentrates	Mixed beverages from powder, often fruit flavors or teas. Examples include Tang and Nestlé Orange-C.
Juice Drinks (up to 24% Juice)	Juices from concentrate that contain up to 24% juice.
Nectars	Juices manufactured from fruit puree or concentrate. Typically contains about 50% juice but can range as low as 25% juice.
RTD Coffee/Tea	Packaged coffee/tea based on brewed coffee/tea or extract with added sugars.
Sports and Energy Drinks	Sports drinks have added sugar but varying electrolyte content. Energy drinks typically have added sugars, B vitamins, and caffeine.
Low Calorie (Diet) Cola Carbonates	Colas with reduced sugar content relative to full sugar brands and may contain artificial sweeteners instead of regular sugar. A branded example is Diet Coke.
Total Bottled Water	Includes plain bottled water such as sparkling, spring, and purified. Total Bottled water category also includes functional bottled water (e.g. Propel fitness water).

Table S4. Consumption per capita and per consumer of beverage type for preschoolers (3-5 years) reported in kilocalories (kcal) (N=958). Regulation status is assigned according to June 2016 cutoffs (first phase). Adjusted means and standard deviations (SD) are presented.

	**kcal per capita**		**% Consumers**	**kcal per consumer**	
**Regulated beverage category**	Mean	SD		Mean	SD
Sweetened flavored water	6	3.3	11.3	55	6.3
Energy and sports drinks	--	--	--	--	--
Carbonated sodas	17	11.8	21.1	71	24.8
Fruit and vegetables drinks	44	20.2	34.0	122	16.0
Coffee and tea	--	--	--	--	--
Dairy-based beverages and dairy subs	109	29.7	55.4	195	29.0
Special formula and nutritional supplements	9	6.5	3.2	278	28.3
Total Regulated beverages	185	59.9	81.2	223	53.6
**Unregulated beverage category**	Mean	SD		Mean	SD
Water and diet flavored water	3	1.2	71.0	4	1.5
Energy and sports drinks	--	--	--	--	--
Carbonated diet sodas	0	0	4.9	0	0
100% fruit and vegetable juices	11	3.1	28.8	38	9.9
Coffee and tea	5	2.3	18.8	24	7.8
Dairy-based beverages and dairy subs	102	23.9	64.3	165	29.0
Special formula and nutritional supplements	19	5.4	8.8	221	46.8
Total Unregulated beverages	139	28.8	94.3	147	27.7
**Total beverages**	324	83.4	100.0	324	83.4

Table S5. Consumption per capita and per consumer of beverage type for adolescents (12-14 years) reported in kilocalories (kcal) (N=767). Regulation status is assigned according to June 2016 cutoffs (first phase). Adjusted means and standard deviations (SD) are presented.

	**kcal per capita**		**% Consumers**	**kcal per consumer**	
**Regulated beverage category**	Mean	SD		Mean	SD
Sweetened flavored water	19	7.1	16.0	113	31.3
Energy and sports drinks	--	--	--	--	--
Carbonated sodas	77	47.3	41.3	172	47.1
Fruit and vegetables drinks	37	14.2	24.5	150	35.3
Coffee and tea	--	--	--	--	--
Dairy-based beverages and dairy subs	41	16.1	23.3	177	26.1
Total Regulated beverages	174	74.0	75.1	227	69.8
**Unregulated beverage category**	Mean	SD		Mean	SD
Water and diet flavored water	2	1.1	63.9	3	1.5
Diet energy and sports drinks	--	--	--	--	--
Carbonated diet sodas	0	0.3	7.2	3	3.9
100% fruit and vegetable juices	5	3.1	20.5	29	14.9
Coffee and tea	41	11.4	54.2	76	18.5

Dairy-based beverages and dairy subs	35	7.1	32.7	109	26.3
Special formula and nutritional supplements	2	1.4	1.2	121	93.3
Total Unregulated beverages	87	15.4	93.9	92	15.5
**Total beverages**	261	85.0	99.5	262	84.4

Table S6. Consumption per capita and per consumer of beverage type for adolescents (12–14 years) reported in mL (*N* = 767). Regulation status is assigned according to June 2016 cutoffs (first phase). Adjusted means and standard deviations (SD) are presented.

	**mL per capita**		**% Consumers**	**mL per consumer**	
**Regulated beverage category**	Mean	SD		Mean	SD
Sweetened flavored water	65	24.3	16.0	388	104.2
Energy and sports drinks	--	--	--	--	--
Carbonated sodas	188	115.6	41.3	422	113.8
Fruit and vegetables drinks	73	29.6	24.5	301	82.7
Coffee and tea	--	--	--	--	--
Dairy-based beverages and dairy subs	63	21.3	23.3	272	30.2
Total Regulated beverages	390	162.5	75.1	509	153.9
**Unregulated beverage category**	Mean	SD		Mean	SD
Water and diet flavored water	193	72.7	63.9	294	83.9
Diet energy and sports drinks	--	--	--	--	--
Carbonated diet sodas	27	8.0	7.2	371	75.0
100% fruit and vegetable juices	15	7.1	20.5	78	35.6
Coffee and tea	213	18.2	54.2	396	22.3
Dairy-based beverages and dairy subs	69	13.0	32.7	213	45.9
Special formula and nutritional supplements	2	2.9	1.2	123	415.6
Total Unregulated beverages	522	86.3	93.9	555	83.3
**Total beverages**	912	196.4	99.5	916	193.1

Table S7. Consumption per capita and per consumer of beverage type for preschoolers (3-5 years) reported in kilocalories (kcal) (N=958). Regulation status is assigned according to June 2019 cutoffs (third phase). Adjusted means and standard deviations (SD) are presented.

	**kcal per capita**		**% Consumers**	**kcal per consumer**	
**Regulated beverage category**	Mean	SD		Mean	SD
Sweetened flavored water	7	3.0	12.5	55	6.3
Energy and sports drinks	--	--	--	--	--
Carbonated sodas	17	11.8	21.1	71	24.8
Fruit and vegetables drinks	44	20.1	34.7	123	16.0
Coffee and tea	--	--	--	--	--
Dairy-based beverages and dairy subs	127	35.6	63.3	208	33.3
Special formula and nutritional supplements	10	6.8	3.9	243	110.8
Total Regulated beverages	205	66.7	85.1	237	59.6
**Unregulated beverage category**	Mean	SD		Mean	SD
Water and diet flavored water	2	0.9	70.7	3	1.1
Energy and sports drinks	--	--	--	--	--
Carbonated diet sodas	0	0	4.9	0	0
100% fruit and vegetable juices	11	3.3	28.3	37	10.3
Coffee and tea	5	2.3	18.8	24	7.8
Dairy-based beverages and dairy subs	84	16.3	52.2	137	21.1
Special formula and nutritional supplements	18	6.0	8.3	203	43.6
Total Unregulated beverages	119	21.9	93.1	127	21.3
**Total beverages**	324	83.4	100.0	324	83.4

Table S8. Consumption per capita and per consumer of beverage type for adolescents (12-14 years) reported in kilocalories (kcal) (N=767). Regulation status is assigned according to June 2019 cutoffs (third phase). Adjusted means and standard deviations (SD) are presented.

	**kcal per capita**		**% Consumers**	**kcal per consumer**	
**Regulated beverage category**	Mean	SD		Mean	SD
Sweetened flavored water	19	7.1	16.0	113	31.3
Energy and sports drinks	--	--	--	--	--
Carbonated sodas	77	47.4	41.5	172	47.1
Fruit and vegetables drinks	37	14.2	24.6	150	35.3
Coffee and tea	--	--	--	--	--
Dairy-based beverages and dairy subs	51	17.5	31.4	181	26.0
Total Regulated beverages	186	73.7	78.2	233	69.6
**Unregulated beverage category**	Mean	SD		Mean	SD
Water and diet flavored water	2	1.1	63.9	3	1.5
Diet energy and sports drinks	--	--	--	--	--
Carbonated diet sodas	0	0.3	7.0	2	4.2
100% fruit and vegetable juices	5	3.2	20.5	28	15.2
Coffee and tea	41	11.4	54.2	76	18.5
Dairy-based beverages and dairy subs	25	4.4	26.6	79	19.6
Special formula and nutritional supplements	1	1.0	0.9	65	111.6
Total Unregulated beverages	75	13.4	93.1	80	14.0
**Total beverages**	261	85.0	99.5	262	84.4

Table S9. Consumption per capita and per consumer of beverage type for preschoolers (3-5 years), reported in milliliters (mL) (N=958). Regulation status is assigned according to June 2016 cutoffs (first phase). Adjusted means and standard deviations (SD) are presented.

	**mL per capita**		**% Consumers**	**mL per consumer**	
**Regulated beverage category**	Mean	SD		Mean	SD
Sweetened flavored water	23	11.9	11.3	198	22.2
Energy and sports drinks	--	--	--	--	--
Carbonated sodas	41	29.6	21.0	174	62.6
Fruit and vegetables drinks	82	37.7	34.0	230	29.5
Coffee and tea	--	--	--	--	--
Dairy-based beverages and dairy subs	186	41.2	58.1	333	30.7
Special formula and nutritional supplements	2	1.3	3.2	48	26.5
Total Regulated beverages	333	95.2	82.1	404	81.5
**Unregulated beverage category**	Mean	SD		Mean	SD
Water and diet flavored water	233	50.9	72.9	325	52.0
Diet energy and sports drinks	--	--	--	--	--
Carbonated diet sodas	7	9.2	4.9	126	64.8
100% fruit and vegetable juices	30	7.9	28.7	106	28.9
Coffee and tea	41	10.2	18.8	216	14.8
Dairy-based beverages and dairy subs	199	30.5	64.3	325	24.8
Special formula and nutritional supplements	7	5.1	8.8	76	44.3
Total Unregulated beverages	518	78.2	94.7	548	72.1
**Total beverages**	852	151.2	100.0	852	151.2

Table S10. Consumption per capita and per consumer of beverage type for preschoolers (3-5 years), reported in milliliters (mL) (*N* = 958). Regulation status is assigned according to June 2019 cutoffs (third phase). Adjusted means and standard deviations (SD) are presented.

	**mL per capita**		**% Consumers**	**mL per consumer**	

Regulated beverage category	Mean	SD		Mean	SD
Sweetened flavored water	25	10.6	11.3	198	22.2
Energy and sports drinks	--	--	--	--	--
Carbonated sodas	41	29.6	21.1	174	62.6
Fruit and vegetables drinks	83	37.0	34.0	231	29.4
Coffee and tea	--	--	--	--	--
Dairy-based beverages and dairy subs	219	52.6	55.4	359	40.2
Special formula and nutritional supplements	2	1.4	3.2	48	26.5
Total Regulated beverages	371	108.2	85.1	430	94.1
**Unregulated beverage category**	Mean	SD		Mean	SD
Water and diet flavored water	231	50.4	71.0	322	51.5
Diet energy and sports drinks	--	--	--	--	--
Carbonated diet sodas	7	9.2	4.9	126	64.8
100% fruit and vegetable juices	28	8.7	28.8	100	30.3
Coffee and tea	41	10.2	18.8	216	14.8
Dairy-based beverages and dairy subs	166	20.1	61.0	272	19.5
Special formula and nutritional supplements	7	5.1	8.3	74	45.3
Total Unregulated beverages	480	68.5	93.1	515	63.2
**Total beverages**	852	151.2	100.0	852	151.2

Table S11. Consumption per capita and per consumer of beverage type for adolescents (12–14 years) reported in mL (*N* = 767). Regulation status is assigned according to June 2019 cutoffs (third phase). Adjusted means and standard deviations (SD) are presented.

	**mL per capita**		**% Consumers**	**mL per consumer**	
**Regulated beverage category**	Mean	SD		Mean	SD
Sweetened flavored water	65	24.3	16.4	388	104
Energy and sports drinks	--	--	--	--	--
Carbonated sodas	189	116.0	41.5	422	113.8
Fruit and vegetables drinks	74	29.5	24.6	301	82.7
Coffee and tea	--	--	--	--	--
Dairy-based beverages and dairy subs	83	25.2	31.4	281	28.4
Total Regulated beverages	414	161.9	78.2	519	153.4
**Unregulated beverage category**	Mean	SD		Mean	SD
Water and diet flavored water	193	72.7	63.9	294	83.9
Diet energy and sports drinks	--	--	--	--	--
Carbonated diet sodas	26	7.7	7.0	366	69.7
100% fruit and vegetable juices	14	7.5	20.5	76	36.7
Coffee and tea	213	18.2	54.2	396	22.3
Dairy-based beverages and dairy subs	49	8.1	26.6	152	33.7
Special formula and nutritional supplements	2	2.9	0.9	312	535.4
Total Unregulated beverages	498	80.6	93.1	533	77.8
**Total beverages**	912	196.4	99.5	916	193.1

Table S12. Sensitivity analysis presenting significant differences in energy consumed from beverage categories on weekdays versus weekends.

**Regulated Sodas – Preschoolers**		
**Beverage category**	**Mean (kcal/capita)**	**95% CI**
Combined	17	15-19
Weekdays	14*	11-16
Weekends	33*	22-44
**Regulated fruit and vegetable drinks - Preschoolers**		
Combined	44	39-48
Weekdays	48*	43-53
Weekends	18*	10-25

Regulated Sodas – Adolescents		
Combined	75	67-84
Weekdays	66*	57-75
Weekends	116*	95-137

*Denotes statistical significance between groups ($p<0.05$).
